# A pilot study of varicella zoster infection among patients with fever and rash in a tertiary care centre

**DOI:** 10.1186/1471-2334-14-S3-P74

**Published:** 2014-05-27

**Authors:** Siddhartha Ojah, Ramya Barani, Padma Srikanth

**Affiliations:** 1 Department of Microbiology, Sri Ramachandra Medical College and Research Institute, Sri Ramachandra University, Porur, Chennai, India

## Background

Varicella zoster virus (VZV) causes mild self limiting illness in children. Older children and adults may present with atypical manifestations. At present VZV vaccine is not included in national immunization schedule. The study was undertaken to determine the presence of VZV infection in patients with acute febrile illness with rash.

## Methods

Blood samples were collected from patients (n=36) with acute febrile illness with rash in a tertiary care hospital. IgM ELISA was performed according to manufacturer’s instructions (IBL International, Germany). Kit standards were used to obtain a standard curve. The results of the samples were interpreted from the standard curve.

## Results

All enrolled patients had fever with rash without evidence of vesicles. Majority, (n=29) were adults and 18 were male. Of the 14 (38.9%) that were positive by IgM ELISA for VZV, 5 (35.7%) belonged to the age group of 19-30 years, 4 (28.5%) were < 18 years and 2 (14.3%) in age group 51-60 years. All patients had normal platelet count except 9, who had mildly deranged platelet count (>1.5 to < 2 lakh /cumm). All sera that were tested were negative for Dengue NS1 antigen, IgM and IgG antibody capture ELISA (Panbio, Australia).

## Conclusion

VZV may present with atypical manifestation. Non progression of rash to vesicle may warrant testing for VZV and other viruses in the absence of positive Dengue serology.

